# Perioperative Management of Scimitar Syndrome Mimicking Total Anomalous Pulmonary Venous Return Posing a Diagnostic Dilemma: A Compilation of Two Cases

**DOI:** 10.7759/cureus.19107

**Published:** 2021-10-28

**Authors:** Gurpreet Panesar, Vishal V Bhende, Tanishq S Sharma, Nirja Patel, Amit Kumar, Bhadra Y Trivedi, Kunal Soni, Kartik Dhami, Deepakkumar V Mehta

**Affiliations:** 1 Cardiac Anaesthesiology, Bhanubhai and Madhuben Patel Cardiac Centre, Shree Krishna Hospital, Anand, IND; 2 Pediatric Cardiac Surgery, Bhanubhai and Madhuben Patel Cardiac Centre, Shree Krishna Hospital, Anand, IND; 3 Cardiac Intensive Care Unit, Bhanubhai and Madhuben Patel Cardiac Centre, Shree Krishna Hospital, Anand, IND; 4 Pediatric Cardiology, Bhanubhai and Madhuben Patel Cardiac Centre, Shree Krishna Hospital, Anand, IND; 5 Radiodiagnosis & Imaging, Pramukhswami Medical College & Shree Krishna Hospital, Bhaikaka University, Karamsad, IND

**Keywords:** atrial septal defect, congenital anomaly, pulmonary arterial hypertension, anomalous pulmonary venous drainage, scimitar syndrome

## Abstract

The low prevalence of scimitar syndrome along with its varied clinical presentation poses a diagnostic dilemma to the treating clinicians. It usually falls under a large spectrum of conditions called venolobar syndrome. Scimitar involves the partial venous drainage of the right lung to the inferior vena cava (IVC). We share our experience of two cases that were diagnosed as partial anomalous pulmonary venous connection/drainage (PAPVC/PAPVD) on echocardiography but CT scan revealed the underlying scimitar syndrome. Perioperative pulmonary arterial hypertension, intraoperative ventilation strategies for managing associated lung hypoplasia, and postoperative right ventricular dysfunction are a few challenges faced in the perioperative period.

## Introduction

With a very low incidence of one to three per 1,00,000 births, scimitar syndrome not only provides a diagnostic dilemma but a challenge in perioperative management. Scimitar syndrome is characterized by the total or partial pulmonary venous return from the right lung to the inferior vena cava or right atrium, commonly associated with right lung hypoplasia, pulmonary artery hypoplasia, anomalous systemic blood supply to the ipsilateral lung, and dextrocardia [1].

## Case presentation

Case 1

**Figure 1:**
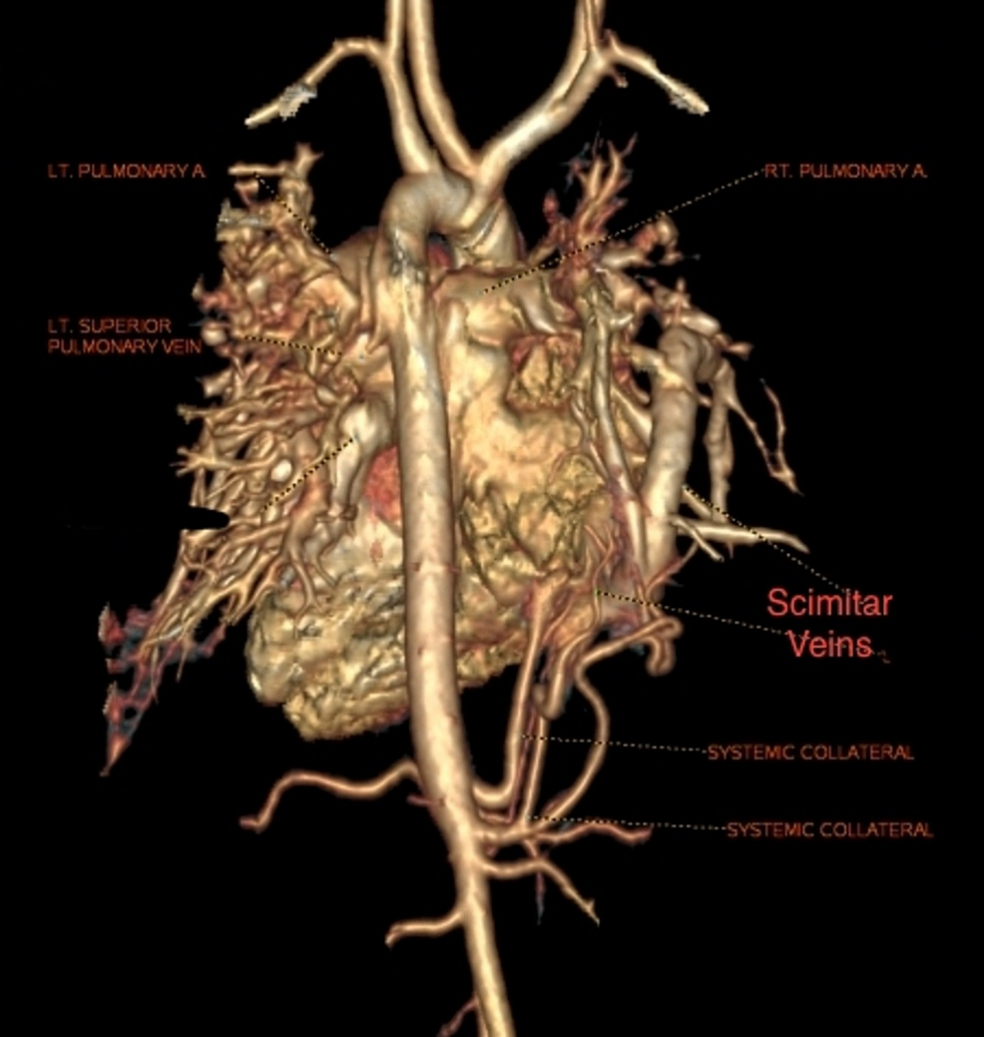
CT cardiac dynamic study volume rendering image showing scimitar veins and systemic collaterals (posterior view)

**Figure 2:**
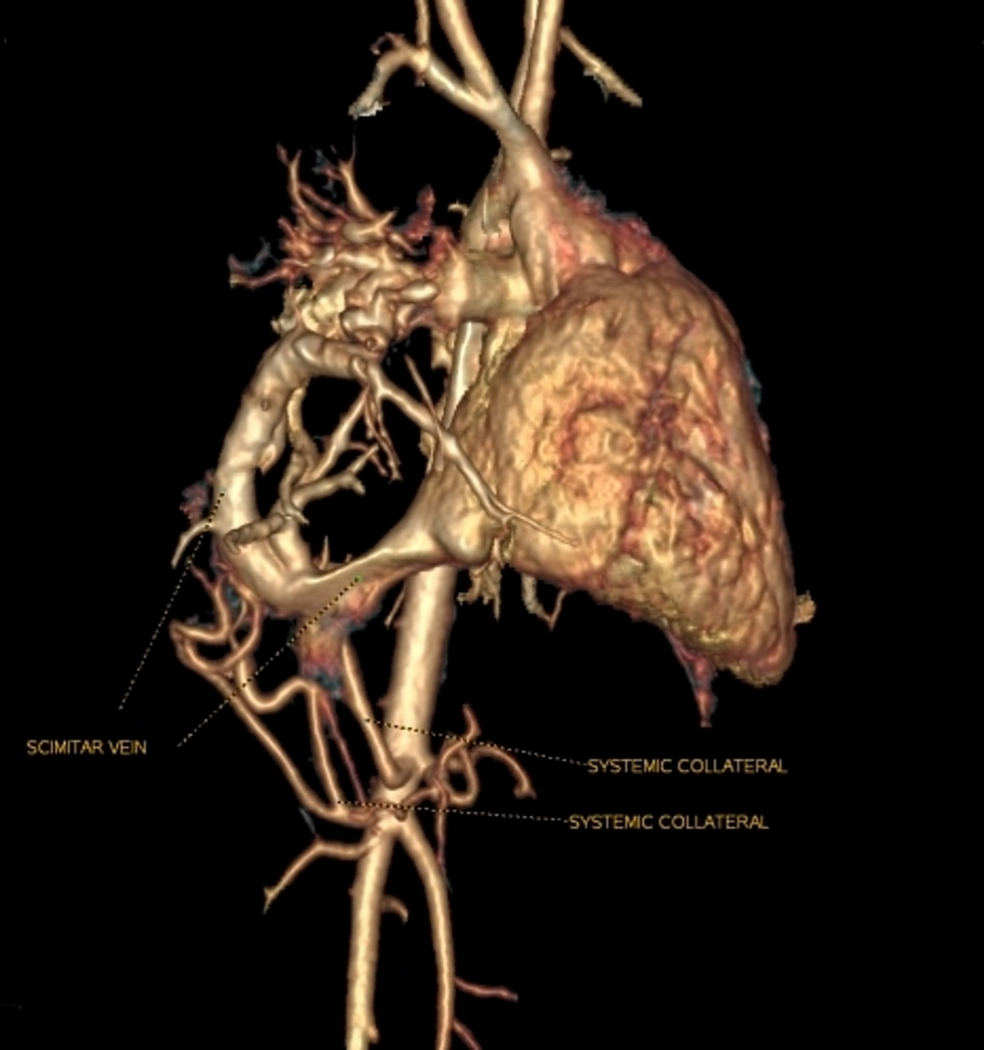
CT cardiac dynamic study volume rendering image showing scimitar veins and systemic collaterals (right lateral view)

This is a case of a one-and-a-half-year-old child weighing 4.53 kgs, who presented to the pediatric cardiac outpatient department with chief complaints of frequent respiratory tract infection and rapid breathing since birth. The child had normal birth history and achieved milestones at the expected age. Echocardiography showed total anomalous pulmonary venous return (TAPVR) with the possibility of infra-cardiac or mixed type drainage of venous return. To gain clarity on the anatomy of pulmonary venous drainage, dynamic cardiac computed tomography (CT) was done (Figures 1-2). CT scan confirmed the inferomedial course of the right superior vein and right inferior pulmonary vein opening in fusiform dilatation of a terminal subdiaphragmatic portion of the inferior vena cava (IVC), which opened in the right atrium. This confirmed the diagnosis of scimitar syndrome associated with an atrial septal defect (ASD) with severe pulmonary arterial hypertension. There was marked dilatation of the right atrium and right ventricle with a cardiothoracic ratio of 10.1 having dextrovert orientation.

**Figure 3:**
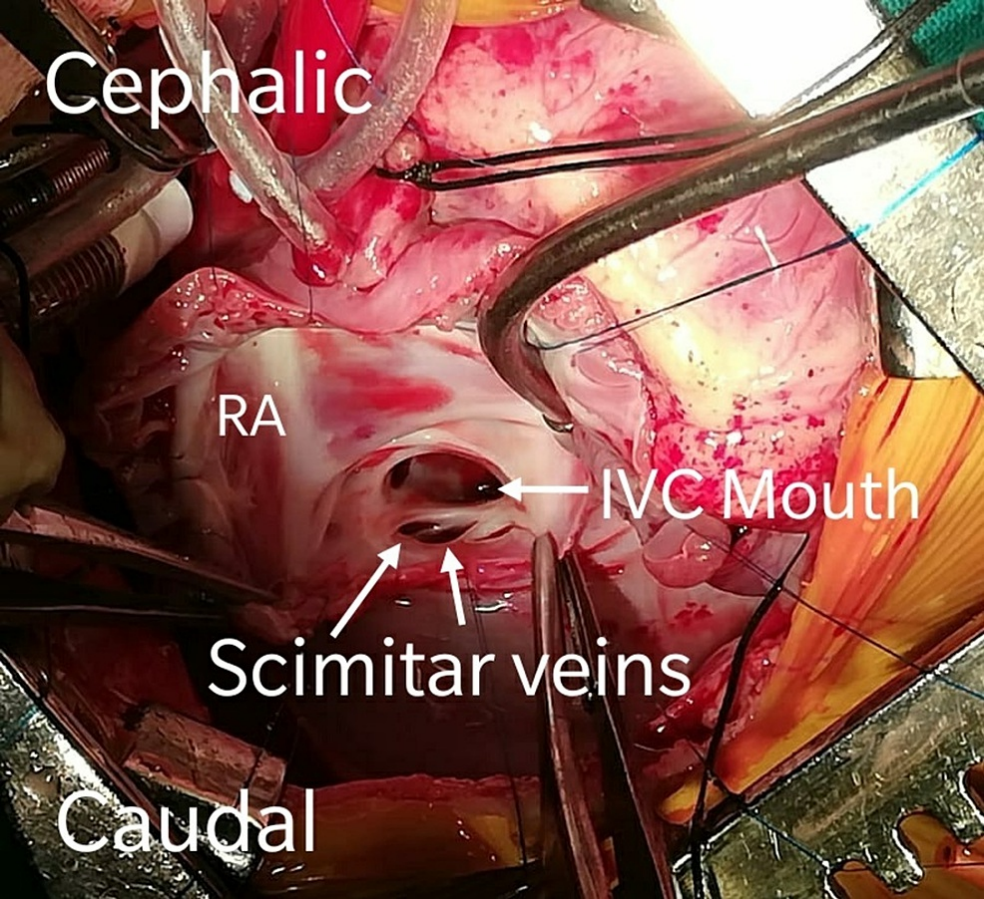
Intra-operative picture of right-sided pulmonary veins draining into the inferior vena cava RA - Right atrium; IVC - Inferior vena cava

With the high-risk written informed consent of relatives and confirmation of the availability of adequate blood products, the child was scheduled for re-routing of right partial anomalous pulmonary venous connection (PAPVC) and ASD closure. Induction was done with midazolam 0.1 mg/kg IV and fentanyl 2 mcg/kg IV followed by vecuronium 0.02 mg/kg IV. Anesthesia was maintained with fentanyl and vecuronium top-up doses and sevoflurane for inhalation agent. Monitoring included pulse oximetry, electrocardiography, end-tidal CO_2_ monitoring, central venous pressure monitoring, invasive arterial monitoring, and temperature monitoring. After opening the sternum, CT findings were confirmed. After the institution of cardiopulmonary bypass (CPB), a longitudinal incision was made on the IVC extending it up to the previous incision of the right atrium (RA) till the whole IVC opening of the right PAPVC was visible (Video 1). Rerouting of the right-sided pulmonary vein at the IVC-RA junction into the left atrium (LA) was carried out by a glutaraldehyde-treated pericardial patch through ASD using 6-0 prolene continuous sutures (Figure 3). RA closure was done in two layers with 6-0 prolene sutures by augmentation of the glutaraldehyde-treated patch at the IVC-RA junction. Weaning off from the bypass required high inotropic support but was uneventful. Post-correction pulmonary artery pressures were less than 2/3rd of the systemic pressure. Epicardial echocardiography demonstrated an unobstructed flow from the right-sided pulmonary venous channel to the left atrium with mild tricuspid regurgitation (TR).

**Video 1:** Intraoperative video of scimitar syndrome Video Credits: Dr. Vishal V. Bhende

In view of high inotropic support and bleeding, the patient was shifted to Cardiac Surgical ICU with an open chest (closed with a blood bag). In the postoperative period, the child remained in a low cardiac output state, i.e. tachycardia, borderline hemodynamics, rising lactate, and high inotropic support. Echocardiography showed moderate right ventricular (RV) dysfunction with good flow from the right-sided pulmonary venous channel to the left atrium. On postoperative day 4, chest closure was done. The patient was hemodynamically stable and so aggressive diuresis and negative peritoneal dialysis were maintained to reduce the third space edema. The child was extubated on postoperative day 10.

Case 2

**Figure 4:**
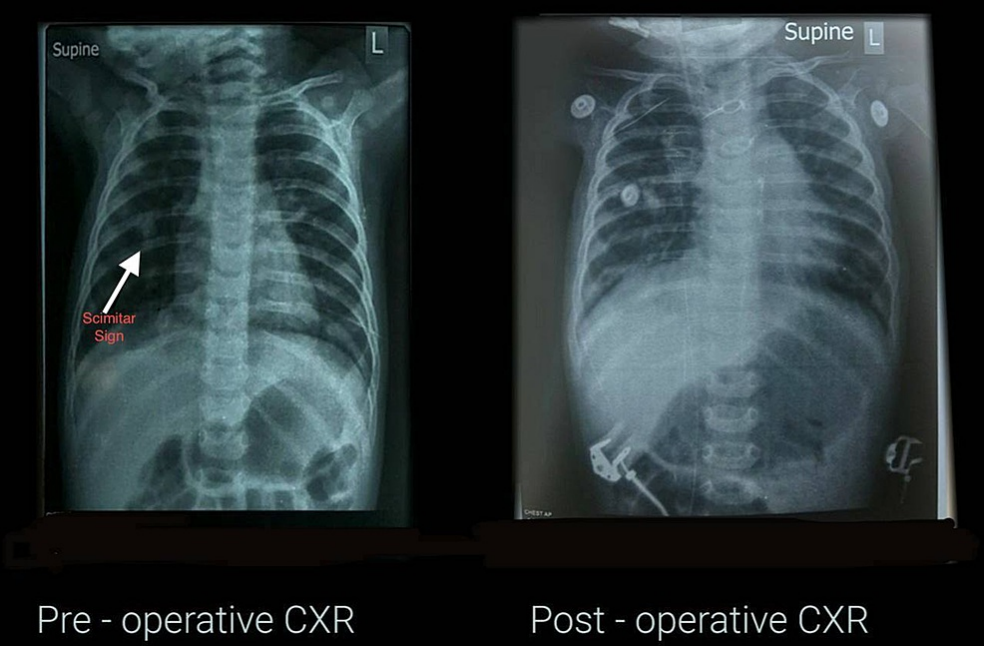
Preoperative (scimitar sign) and postoperative chest radiograph

This is a case of a five-month-old, 4.7 kg child who presented with a history of recurrent lower respiratory tract infections (LRTI). Echocardiography findings suspected scimitar syndrome and so the child was further evaluated with a cardiac CT, which confirmed the finding of a large anomalous right pulmonary vein (scimitar vein) draining into the IVC. With all written informed high-risk consent, rerouting of the right PAPVC was done along with ASD closure under TCA. Weaning from CPB required inotropes but was uneventful. The patient was shifted to ICU with an open chest. On the first postoperative day, chest closure was done. On the fifth postoperative day, the child was extubated. Postoperative echocardiography confirmed the absence of any pulmonary vein drainage in IVC and intact atrial septum with good biventricular function. On the eleventh postoperative day, the child was discharged. A preoperative and a postoperative chest radiograph were also taken (Figure 4).

## Discussion

**Figure 5:**
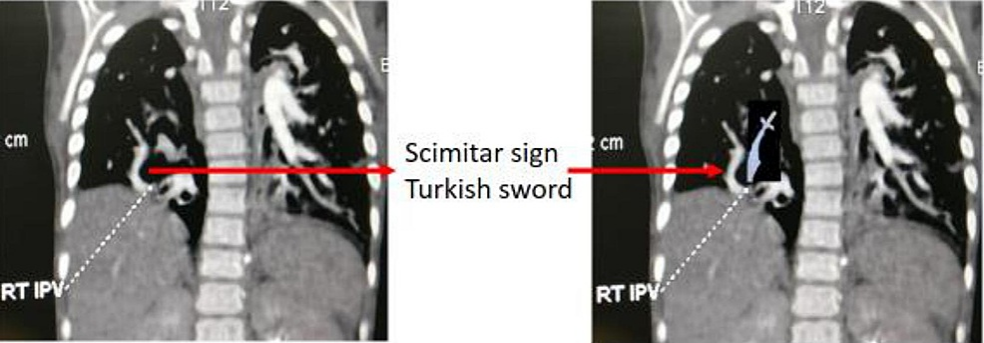
Turkish sword appearance in CT scan Image credits - Dr. Deepakkumar V. Mehta and Dr. Nirja Patel

Scimitar syndrome, a rare congenital cardiac disease with a high perioperative mortality rate [1-2], is named so because the anomalous venous return from the right lung entering the right atrium or vena cava resembles the shape of the Turkish blade "scimitar" [3]. It is seen on a CT scan as a long and curved shadow at the right edge of the heart (Figure 5).

Commonly associated cardiac anomalies with scimitar syndrome are the absence of pericardium and ASD in 60-70% of cases and ventricular septal defect (VSD), coarctation of the aorta, tetralogy of Fallot in the other 25% [3-6]. Anomalous pulmonary venous return of the heart imposes significant volume overload on the right-sided heart cavities because of the left to right shunting of the blood. Pulmonary vein hypoplasia and stricture of PV, if present, aggravate the pressure on the right heart chambers and may lead to persistent pulmonary hypertension [4]. Clinical presentation of scimitar syndrome in the neonatal period is secondary to the severity of underlying pulmonary hypertension that may present as recurrent or refractory respiratory tract infection or respiratory failure [4,7]. Prognosis depends not only on pulmonary hypertension but also on the associated cardiac abnormalities and general condition of the child.

Echocardiography is diagnostic only in 10% of the cases by identifying the scimitar vein [3]. In the rest of the cases, computed tomography and magnetic resonance imaging help confirm the diagnosis. Cardiac catheterization and angiography define the anatomy and severity of underlying pulmonary hypertension and so guide the surgical plan.

Children who have recurrent pulmonary infections, refractory pulmonary hypertension, and signs and symptoms of heart failure are a candidate for surgical correction [2,8]. Surgical repair of scimitar syndrome consists of direct anastomosis of the scimitar vein into the back of the left atrium when the atrial septum is intact or division and reimplantation of the pulmonary vein into the right atrium with baffled flow through an existing or created ASD, which was proposed by Schumaker and Judd [6]. Dissociated anomalies like pulmonary sequestration require lobectomies, anomalous systemic flow requires interruption through embolization or direct ligation, and atrial septal defect needs closure.

A detailed pre-anesthetic review to assess the general condition of the child along with the severity of associated anomalies is important before taking the patient to the operation theatre. Intraoperative monitoring with CO_2_, ECG, two invasive arterial lines, arterial blood gases (ABG), central venous pressure (CVP), and temperature monitoring is mandatory to pick up warning signs at the earliest. The goals of perioperative management are to ensure an adequate plane of anesthesia and proper analgesia to avoid any increase in pulmonary artery pressure but, at the same time, to avoid any myocardial depression, thus ensuring optimum heart function and stable hemodynamics [3]. Inhalation agents are less effective due to the presence of associated pulmonary hypoplasia.

Use of pressure-controlled ventilation with low tidal volume 6-8 ml/kg is preferred keeping plateau pressure less than 30 cmH2O given the underlying hypoplasia. Perioperatively, all precipitating factors for pulmonary hypertension should be avoided, i.e. hypoxia, hypercarbia, hypovolemia, metabolic and respiratory acidosis, and hypothermia [3,6]. If pulmonary hypertension occurs, it should be treated with hyperventilation with 100% oxygen, correction of hypovolemia with volume therapy and appropriate blood products, and use of pulmonary vasodilators like inhaled NO. For the management of hypotension, vasoconstrictors like noradrenaline can be started to improve systemic vascular resistance (SVR) and inotropic agents like milrinone or dobutamine are helpful by increasing inotropic function, simultaneously reducing pulmonary vascular resistance (PVR) [9]. Good analgesia, inotropic support, meticulous hemodynamic monitoring, and respiratory care should continue in postoperative intensive care. Table 1 shows the intraoperative and postoperative parameters of both cases.

**Table 1:** Intraoperative and postoperative parameters of both cases

INTRAOPERATIVE AND POSTOPERATIVE PARAMETERS OF BOTH THE CASES		
Parameters	Case 1	Case 2
Age	14 months	5 months
Weight	4.5 kg	4.7 kg
Cross clamp time	138 minutes	167 minutes
Cardiopulmonary bypass time	245 minutes	250 minutes
Total circulatory arrest time	63 minutes	93 minutes
Time of extubation	10^th^ day	6^th^ day
Ventilatory hours	266 hours	135 hours
Hospital stay	29 days	16days

## Conclusions

Scimitar syndrome is a constellation of congenital cardiac and pulmonary disorders. Proper diagnosis with imaging and cardiac catheterization is mandatory for deciding the surgical plan. Managing perioperative pulmonary hypertension and optimizing hemodynamics and heart function, along with good analgesia in postoperative periods, are important perioperative pillars in management.
